# Very-Low-Calorie Ketogenic Diet as a Safe and Valuable Tool for Long-Term Glycemic Management in Patients with Obesity and Type 2 Diabetes

**DOI:** 10.3390/nu13030758

**Published:** 2021-02-26

**Authors:** Eleonora Moriconi, Elisabetta Camajani, Andrea Fabbri, Andrea Lenzi, Massimiliano Caprio

**Affiliations:** 1 Laboratory of Cardiovascular Endocrinology, IRCCS San Raffaele Pisana, 00166 Rome, Italy; eleonoramoriconi87@gmail.com; 2 PhD Programme in Endocrinological Sciences, Sapienza University of Rome, 00161 Rome, Italy; elisabetta.camajani@uniroma5.it; 3 Department of Human Sciences and Promotion of the Quality of Life, San Raffaele Roma Open University, 00166 Rome, Italy; 4 Division of Endocrinology, CTO Andrea Alesini Hospital, ASL Roma 2, Department of Systems Medicine, University of Rome “Tor Vergata”, 00133 Rome, Italy; andrea.fabbri@uniroma2.it; 5 Section of Medical Pathophysiology and Endocrinology, Department of Experimental Medicine, Sapienza University of Rome, 00161 Rome, Italy; andrea.lenzi@uniroma1.it

**Keywords:** VLCKD, obesity, diabetes remission, weight-loss, eating behavior

## Abstract

Obesity-related type 2 diabetes represents one of the most difficult challenges for the healthcare system. This retrospective study aims to determine the efficacy, safety and durability of a very-low-calorie ketogenic diet (VLCKD), compared to a standard low-calorie diet (LCD) on weight-loss, glycemic management, eating behavior and quality of life in patients with type 2 diabetes (T2DM) and obesity. Thirty patients with obesity and T2DM, aged between 35 and 75 years, who met the inclusion criteria and accepted to adhere to a VLCKD or a LCD nutritional program, were consecutively selected from our electronic database. Fifteen patients followed a structured VLCKD protocol, fifteen followed a classical LCD. At the beginning of the nutritional protocol, all patients were asked to stop any antidiabetic medications, with the exception of metformin. Data were collected at baseline and after 3 (T1) and 12 (T2) months. At T1 and T2, BMI was significantly reduced in the VLCKD group (*p* < 0.001), whereas it remained substantially unchanged in the LCD group. HbA1c was significantly reduced in the VLCKD group (*p* = 0.002), whereas a slight, although not significant, decrease was observed in the LCD group. Quality of life and eating behavior scores were improved in the VLCKD group, whereas no significant changes were reported in the LCD group, both at T1 and T2. At the end of the study, in the VLCKD group 26.6% of patients had stopped all antidiabetic medications, and 73.3% were taking only metformin, whereas 46.6% of LCD patients had to increase antidiabetic medications. The study confirms a valuable therapeutic effect of VLCKD in the long-term management of obesity and T2DM and its potential contribution to remission of the disease.

## 1. Introduction

In 2017, approximately 462 million individuals were affected by type 2 diabetes worldwide. Globally, the prevalence of type 2 diabetes is projected to increase by 2030, with an alarming rise across all regions of the world [1].

Although genetic predisposition plays a key role in individual susceptibility to T2DM, high prevalence of obesity, population aging and greater longevity contribute to this growing pandemic [2].

Specifically, the worldwide prevalence of obesity tripled between 1975 and 2016. In 2016, more than 1.9 billion adults were overweight and over 650 million adults were obese. It has been estimated that across the world about 13% of adults (11% of men and 15% of women) were obese in 2016 [3].

Increased body mass index (BMI) represents a major risk factor for noncommunicable diseases such as cardiovascular disease, musculoskeletal disorders and diabetes [4,5]. The term “diabesity” highlights the key role of obesity in the pathogenesis of T2DM and it has been estimated that the probability of developing diabetes grows by 4.5% for every kilogram increase in body weight [6].

The presence of obesity and T2DM has been shown to represent a risk factor for the development and severity of SarS-Cov2 infection [7,8]. The association between obesity and poor COVID-19 prognosis may be attributable to the fact that obesity is known to impair immune response to viral infections, to induce a chronic low-grade inflammatory state and to increase oxidative stress [9,10,11]. 

Previous meta-analyses have demonstrated the beneficial effects of a very-low-calorie ketogenic diet (VLCKD) on weight loss in obese patients [12,13,14]. However, there is still scarce evidence for the use of VLCKD as a safe and effective tool for the management of T2DM in the long term. Goday et al. showed that, as part of a weight loss program that included lifestyle and behavioral changes, a 12-week VLCKD resulted in higher adherence and satisfaction compared to a low-calorie diet [15]. VLCKD use has also been linked to the recovery of first phase of insulin secretion and, consequently, to a significant reduction in the need for glucose-lowering agents, including insulin [16]. Recently, the American Diabetes Association (ADA) included the use of VLCKD as a viable therapeutic option for the treatment of T2DM patients with obesity [17]. 

This retrospective study was designed to evaluate safety, efficacy and durability of a 12 month VLCKD regimen on body weight and glycemic control in a selected group of patients with T2DM and obesity. Several metabolic parameters were collected at the beginning of the VLCKD regimen and after 3 and 12 months of intervention. The results were compared to a similar group of subjects with T2DM and obesity who were prescribed a 12 month standard low-calorie diet regimen (LCD). The effect of the VLCKD regimen on T2DM remission rate and need for pharmacological therapy was also evaluated at 12 months and compared to that of the LCD regimen. Changes in quality of life and eating behaviors were assessed through the 36-Item Short Form Health Survey questionnaire (SF-36) and The Three-Factor Eating Questionnaire (TFEQ-R18). 

## 2. Materials and Methods

### 2.1. Subjects

Patients included in this retrospective observational study were consecutively selected among those attending the Diabetes Unit of the Division of Endocrinology, CTO Andrea Alesini Hospital, ASL Roma 2, Rome, Italy, between April 2018 and June 2020, who followed the inclusion criteria and accepted to adhere to a VLCKD or a LCD nutritional program. Importantly, patients were free to choose either a VLCKD or LCD nutritional program, after a careful explanation of the two dietary protocols. 

Upon admission, all patients signed an informed consent form in accordance with the General Data Protection Regulation (GPDR, 2016/679). Antidiabetic therapy (with the exception of metformin) was stopped concomitantly with the beginning of dietary regimens (VLCKD or LCD). Patients were examined once every month during the first 6 months from the beginning of the intervention (T0), then after 9 and 12 months from T0. Anonymized data were obtained from electronic medical records (Smart Digital Clinic–Meteda SRL, San Benedetto del Tronto, Italy) at baseline (T0), 3 months (T1) and 12 months (T2) after the beginning of the nutritional regimen. Inclusion criteria were: T2DM, defined as HbA1c > 6.5% (48 mmol/L) or fasting plasma glucose (FPG) level ≥ 126 mg/dL (7 mmol/L), plasma glucose level ≥ 200 mg/dL (11.1 mmol/L) 2 h after the ingestion of 75 mg of glucose, or the use of glucose-lowering medications; fairly good metabolic control (HbA1c < 8.5%) at the beginning of intervention, allowing for suspension of oral therapy (except for metformin); BMI ≥ 27 kg/m^2^; nutritional follow-up for at least 12 months. Exclusion criteria: current treatment with steroids or antineoplastic agents; type 1 diabetes; β-cell failure in T2DM, insulin therapy or concomitant use of sodium/glucose cotransporter 2 (SGLT2) inhibitors (risk for euglycemic diabetic ketoacidosis); diabetes with severe chronic complications such as nephropathy, retinopathy, and neuropathy; psychiatric disturbances; pregnancy; lactation; kidney failure (estimated glomerular filtration rate (eGFR) < 60 mL/min); liver failure; heart failure (NYHA III-IV); respiratory failure; planned surgeries; unstable angina or cardiac arrhythmias; recent stroke or myocardial infarction (<12 months) [16,17,18]. Antidiabetic medications could be modified throughout the study, according to HbA1c levels and plasma glucose monitoring.

### 2.2. Laboratory Assay and Anthropometric Parameters

Fasting glycemia, HbA1C, lipid profile, electrolytes, liver enzymes, and renal function were measured. Glomerular Filtration Rate (GFR) was calculated with the Chronic Kidney Disease Epidemiology Collaboration (CKD-EPI) equation [19]. Ketosis was confirmed by β-Hydroxybutyrate capillary blood detection by using a portable meter (GlucoMen LX Sensor, A. Menarini Diagnostics, Neuss, Germany; sensitivity <0.2 mmol/L). The threshold value for nutritional ketosis is blood ketone levels of 0.5 mg/dL [20]. 

Anthropometric measurements were obtained from each patient after a 12 h overnight fast and while wearing a hospital gown. Body weight (kg) was measured to the nearest 0.01 kg, using an accurate balance scale (Invernizzi, Rome, Italy). Height (m) was measured using a stadiometer to the nearest 0.1 cm (Invernizzi, Rome, Italy). BMI was calculated according to Quetelet Index (calculated as body weight divided by height squared (kg/m^2^)). Waist and hip circumferences (cm) were taken using a flexible steel metric tape to the nearest 0.5 cm. Waist circumference was measured at the midpoint between the lower rib margin and the iliac crest. Hip circumference was measured at the greatest posterior protuberance of the buttocks [21]. Systolic and diastolic blood pressure was measured at baseline and at all timepoints of the study.

### 2.3. Quality of Life

The 36-Item Short Form Health Survey questionnaire (SF-36) is an effective measure of Health-Related Quality of Life. The SF-36 measures eight scales: physical functioning (PF), role—physical (RP), bodily pain (BP), general health (GH), vitality (VT), social functioning (SF), role—emotional (RE), and mental health (MH). Each scale score ranges 0–100, where a value of 100 suggests absence of disability. Component analyses showed that there are two distinct concepts measured by the SF-36: a physical dimension, represented by the Physical Component Summary (PCS), and a mental dimension, represented by the Mental Component Summary (MCS). All scales contribute in different proportions to the scoring of both PCS and MCS measures [22]. The Three-Factor Eating Questionnaire (TFEQ) is a well-validated questionnaire for measuring eating behaviors. It investigates three eating-related constructs: dietary restraint, disinhibition, and susceptibility to hunger [23]. Karlsson et al. revised the test to a 18-item questionnaire, the TFEQ-R18 [24]. The results were collected in three areas: cognitive restraint (6 items), uncontrolled eating (9 items) and emotional eating (3 items). According to Lauzon et al., each item was scored from 1 to 4 and the scores were added together to obtain scores for cognitive restraint (range score 6–24), uncontrolled eating (range score 9–36) and emotional eating (range score 3–12) [25]. Restraint refers to the individual ability to restrict food intake. Uncontrolled eating refers to overeating and to the inability to control food intake. Emotional eating refers to propensity to eat in order to feel better [26]. Finally, adherence to the diet was investigated with a nonvalidated questionnaire, which was adapted from the Mediterranean Diet Score [27], indicating scarce adherence when score ranges from 0–5, moderate adherence when score ranges from 5–9 and good adherence when score ranges from 9–14. 

### 2.4. Nutritional Intervention

All patients who were willing to adhere to a strict dietary regimen including nutritional supplements and replacement meals, and did not present contraindications to VLCKD, underwent a multiple-phase VLCKD protocol with the use of replacement meals (Therascience, New Penta SRL or Pronokal Group, each brand containing comparable amounts of calories and similar macronutrient composition) and were strictly followed by a nutrition specialist at the Diabetes Center.

In the first phase (45 days) total daily energy intake was < 800 kcal, with a protein daily intake between 1.2 and 1.5 g per kg of ideal body weight to prevent lean mass loss. During this first phase, patients ate four or five replacement meals per day, according to their specific nutritional needs. In the second phase (45 days), one and subsequently two replacement meals were replaced with conventional food containing proteins (meat, fish, eggs, soy) at lunch and/or dinner. During the first two phases, carbohydrate intake was drastically restricted to induce ketosis and lipid intake was very low and mostly derived from olive oil (≈20 g per day). Recommended water intake was at least 2.5 lt/day. To avoid micronutrient deficiencies, mineral and vitamin supplements were recommended and only erythritol or steviol glycosides were allowed as sweeteners [28]. The maximal duration of the first two phases (ketosis phases) was 3 months (T1). The length of these phases was personalized according to the weight loss target [16].

In the subsequent phases, caloric daily intake was gradually increased and a gradual carbohydrate reintroduction was carried out, starting with low glycemic index carbohydrates. At the end of 6 months, all patients had reintroduced all types of carbohydrates (fruits, dairy products, legumes, bread and cereals). From months 6 to 12, patients followed a balanced maintenance diet, with a daily calorie intake between 1500 and 2000 kcal, according to the patient’s metabolic needs. It was essential during diet protocol to promote a gradual and personalized introduction of physical activity and a healthy lifestyle. The entire nutritional protocol lasted for at least 12 months (T2).

The LCD was based upon a daily reduction in energy intake of 500–1000 kcal, compared to each individual’s basal metabolic rate (estimated via the Harris–Benedict equation). Macronutrient dietary composition consisted on a daily intake of 30% of calories coming from fat (saturated fat <7% kcal/day; polyunsaturated fatty acids, 10–20% kcal/day and monounsaturated fatty acids, 10–20% kcal/day; cholesterol consumption <300 mg/day), 20– 25% from protein and 45–50% from carbohydrates. Fiber daily intake was 25–30 g. Sodium daily intake was <5 g. The nutritional plan consisted of five daily meals, according to the Mediterranean nutritional approach. The protein source was mainly represented by legumes, eggs and fish, whereas whole grains, fruits and fresh vegetables represented the main source of carbohydrates [29]. The low-calorie diet used in our protocol was not isocaloric with the VLCKD. Daily total calorie intake as well as macronutrient composition was assessed through MètaDieta software version 4.1 (Meteda SRL, San Benedetto del Tronto, Italy).

### 2.5. Statistical Analysis

Thirty consecutive patients who satisfied inclusion and exclusion criteria for VLCKD and LCD in the planned period were included in this retrospective observational study. All data considered for statistical analysis were retrieved from existing clinical records. Results are presented as mean ± standard deviation (SD) or standard error (SE). Normality was assessed with the Kolmogorov–Smirnov test. Independent Samples *t* Tests were calculated for each variable with a normal distribution to compare metabolic and anthropometric values in patients following VLCKD versus those following LCD. Within each diet group, paired *t* tests were used to test whether the changes from baseline to 3 and 12 months were significantly different from zero. Mean values of anthropometric and biochemical parameters at baseline, T1 and T2 in VLCKD and LCD groups were compared using *t*-paired test. Bonferroni post hoc analysis was used to compare parameters at different timepoints, and the new level of statistical significance depended upon the number of comparisons. Statistical analyses were performed using the Statistical Package for Social Sciences (SPSS) software (version 20, IBM, Armonk, New York, NY, USA).

## 3. Results

Thirty patients who followed the entire nutritional protocol, and whose data were complete, were selected for the study; fifteen patients followed a VLCKD, fifteen followed a LCD regimen and both groups underwent the same assessments and follow-up throughout the course of the study. Each group was composed of seven females and eight males, with a mean ± SD age of 60.5 ± 10.2 years and 64.4 ± 8.8 years, respectively. The mean weight at baseline was 111.6 ± 19.8 in the VLCKD group and 91.6 ± 18.7 in the LCD group, whereas mean BMI was 39.5 ± 6.0 kg/m^2^ and 32.2 ± 4.3 kg/m^2^, respectively. The duration of diabetes was similar between groups (2.53 ± 1.19 years in the VLCKD group and 2.47 ± 1.36 years in the LCD group). The baseline anthropometric measures (weight, BMI, WC and HC) were significantly higher in the VLCKD group, whereas WHR was not different between the two groups, with a predominantly abdominal fat distribution observed in both groups (WHR 1.00 ± 0.11 in VLCKD group and 0.97 ± 0.05 in LCD group, Table 1). Baseline diastolic and systolic blood pressure, and biochemical parameters were similar between the two groups.

Antidiabetic medications taken at baseline and at the end of the nutritional protocol in VLCKD and LCD groups are reported in Table 2. At baseline, in the VLCKD group, only one patient was not taking any antidiabetic medication and was following a hypocaloric diet. All other patients who entered the VLCKD protocol were taking metformin or metformin in combination with sulphonylurea, GLP-1 agonist, SGLT2-inhbitors or DPP4-inhibitors. At T2, 4 patients (26.6%) in the VLCKD group had stopped all antidiabetic medications, including metformin, whereas 11 patients (73.3%) were taking only metformin. Mean number of antidiabetic medications was significantly decreased in the VLCKD group compared to the LCD group. In fact, at baseline, all patients were taking only metformin in the LCD group, whereas 7 patients (46.6%) had to increase antidiabetic therapy at T2.

In the VLCKD group, a significant weight-loss was observed at 3 (8.5% from baseline, *p* = 0.000) and 12 months (11.5% from baseline, *p* = 0.000), whereas no significant weight change was observed in the LCD group (*p* = 0.706 at T1, *p* = 0.623 at T2). Anthropometric measures (BMI, WC and HC) were significantly reduced at T1 and T2 in VLCKD patients (BMI T0-T1: 39.5 ± 6.0 vs. 35.9 ± 5,3, *p* = 0.000; BMI T2: 34.8 ± 4.04, *p* = 0,000 vs. T0; WC T0-T1: 118.2 ± 9.0 vs. 113,8 ± 7.5, *p* = 0.000; WC T2: 114.5± 6,68, *p* = 0.002 vs. T0; HC T0-T1: 121.6 ± 16.4 vs. 118.1 ± 15.2, *p* = 0.008; HC T2: 117.1 ± 13.9, *p* = 0.006 vs. T0), whereas these parameters remained substantially unchanged in the LCD group (Table 3). We did not observe a significant reduction in waist–hip ratio (WHR), probably as result of an homogenous distribution of weight loss across all body fat compartments. Systolic blood pressure was significantly decreased at T1 and T2 in VLCKD group (SysBP T0-T1: 143.2 ± 16.3 vs. 134.6 ± 10,7, *p* = 0.001; SysBP T2: 129.4 ± 10.0, *p* = 0.002), whereas a significant reduction of SysBP was only observed at T1 in the LCD group (SysBPT0-T1: 136.4 ± 12.3 vs. 133.4 ± 12.9, *p* = 0.024; SysBPT2: 138.8 ± 7.9, *p* = 0.486). Diastolic blood pressure was decreased only at 12 months (T2) in the VLCKD group (*p* = 0.007), whereas it remained unchanged in the LCD group. Total cholesterol decreased in the VLCKD group at all timepoints (Tot chol T0-T1: 205.7 ± 35.1 vs. 178.5 ± 35.7, *p* = 0.001; Tot chol T2: 160.1 ± 40.1, *p* = 0.001), although no significant changes were observed in HDL cholesterol and LDL cholesterol (except for T1 for LDL cholesterol, *p* = 0.010). In the LCD group, the lipid profile remained substantially unchanged. Fasting plasma glucose was reduced at T1 (*p* = 0.024) and T2 (*p* = 0.009) in the VLCKD group; in the LCD group, fasting plasma glucose was unchanged at T1 (*p* = 0.198) and was numerically lower at T2 (*p* = 0.071), although the decrease was not statistically significant. HbA1c was significantly reduced in the VLCKD group at both timepoints, whereas minimal and not statistically significant changes were observed in the LCD group. Finally, creatinine and transaminases were unchanged in both groups at all timepoints (Table 3). There was no clear evidence of sex differences in any of the outcomes within each diet group; however, a subgroup analysis has not been performed due to the small number of patients.

Mean weight loss from baseline (T0) to T1 (i.e., the end of the ketosis phase for the VLCKD group) was 9.51 ± 7.13 Kg in the VLCKD group, whereas it was 0.31 ± 3.08 kg in the LCD group. At T2, mean weight loss was 12.93 ± 8.84 and 0.58 ± 4.18 kg in the VLCKD and LCD groups, respectively. At T1, HbA1c was decreased by 0.69 ± 0.65% in the VLCKD group and by 0.42 ± 0.01% in the LCD group (difference not statistically significant). At T2 the change in HbA1c from baseline was 0.61 ± 0.54% in the VLCKD group and 0.13 ± 0.76% in the LCD group (*p* = 0.070). In both groups, HbA1c was slightly increased between T1 and T2, probably due to a lower adherence of patients during the maintenance phases of both nutritional regimens. All data mentioned above are reported in Table 4.

**Figure 1:**
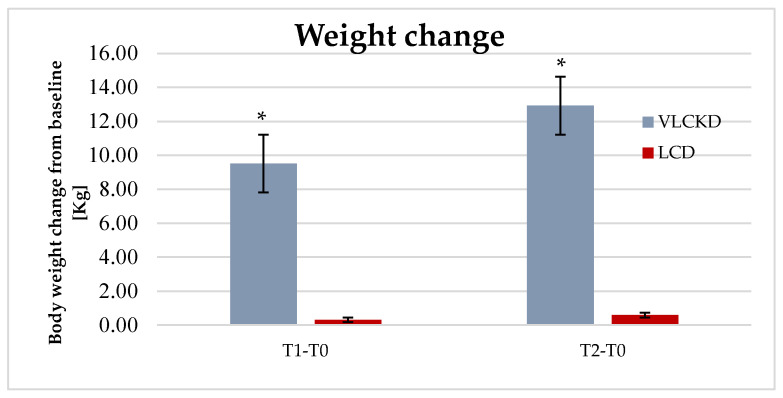
Mean weight loss (± SE) at T1 (3 months) and T2 (12 months) in VLCKD and LCD groups. * *p* < 0.001.

**Figure 2:**
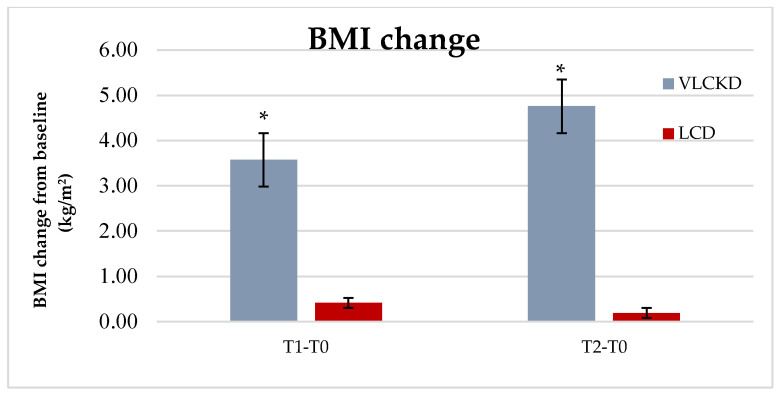
Mean BMI (±SE) reduction at T1 (3 months) and T2 (12 months) in VLCKD and LCD groups. * *p* < 0.001.

Graphical representation of mean weight loss and BMI reduction (±SE) throughout the entire study in the VLCKD and LCD groups is shown in Figure 1 and Figure 2.

**Figure 3:**
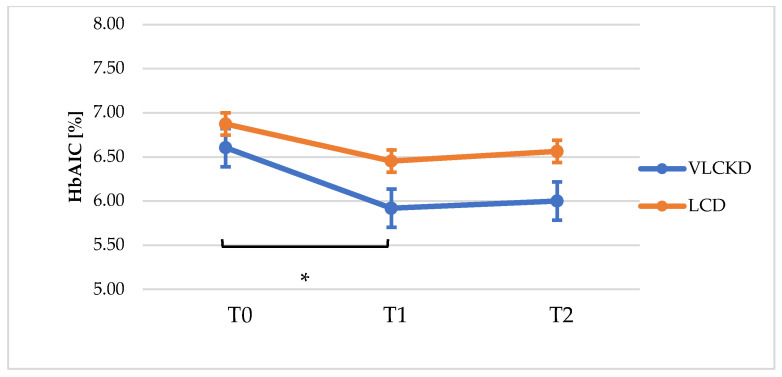
Kinetics of HbA1c values throughout the study (T0, T1 and T2). * *p* < 0.005.

Figure 3 shows the mean variation in HbA1c (expressed as percentage values) at baseline, T1 and T2, in both the VLCKD and LCD groups.

**Figure 4:**
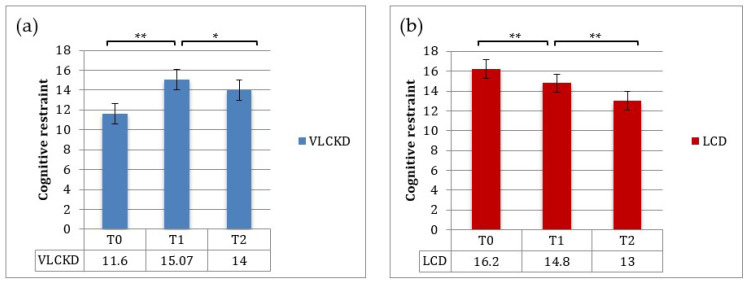
Mean scores (±SE) for cognitive restraint at baseline, T1 and T2: (**a**) VLCKD group; (**b**) LCD group. * *p* < 0.01; ** *p* < 0.001.

**Figure 5:**
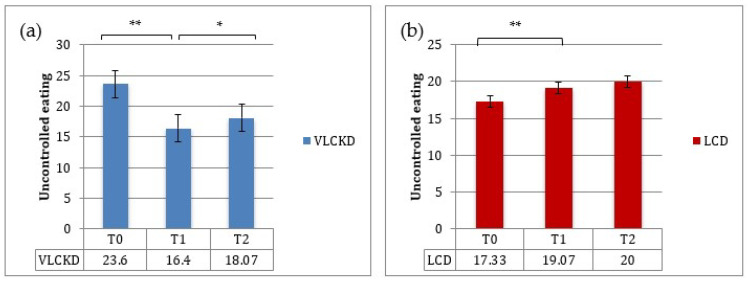
Mean scores (±SE) for uncontrolled eating at baseline, T1 and T2: (**a**) VLCKD group; (**b**) LCD group. * *p* < 0.05; ** *p* < 0.001.

**Figure 6:**
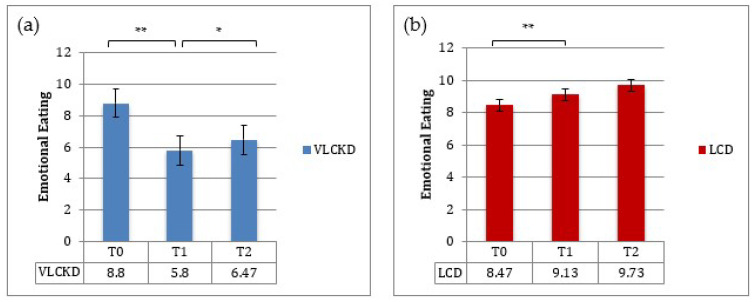
Mean scores (±SE) for emotional eating at baseline, T1 and T2: (**a**) VLCKD group; (**b**) LCD group. * *p* < 0.05; ** *p* < 0.001.

The TFEQ-18 test was administered to patients at baseline and at 3 and 12 months. Baseline mean values for the “cognitive restraint” score (i.e., the degree of cognitive control in daily food intake, score ranging between 6–24) and for the “uncontrolled eating” score (i.e., loss of control in food intake, score ranging between 9–36) were 11,6 ± 4,48 and 23.6 ± 6.42 in the VLCKD group and 16.2 ± 4.46 and 17.3 ± 5.19 in the LCD group. The baseline mean score for “emotional eating” (i.e., the susceptibility to internal or external hunger signs, score ranging between 3–12) was 8.8 ± 1.93 and 8.47 ± 2.13 in the VLCKD and LCD groups, respectively. At T1, the cognitive restraint score increased (*p* < 0.01), whereas both the uncontrolled eating and emotional eating scores decreased (*p* < 0.001) in the VLCKD group (Figure 4). Patients assigned to the LCD showed a significant increase in uncontrolled eating and emotional eating scores (*p* < 0.001) at 3 months (T1) (Figure 5 and Figure 6).

Between T1 and T2, uncontrolled eating and emotional eating scores slightly increased in both groups; this was probably due to a lower adherence to the diet regimen after the first timepoint of observation (Figure 5 and Figure 6).

The mean values for each scale of the quality of life assessment were lower at baseline in the VLCKD group compared to the LCD group. In fact, the mean scores in VLCKD group were lower than 50 (except for role emotional), suggesting a poor quality of life and major disability, mostly due to a more severe degree of obesity.

In the LCD group, the lowest scores were observed in the physical functioning, vitality, social functioning and role—emotional areas, suggesting that obesity limited daily activities (dressing, walking, hygiene), reduced energy and increased fatigue, and induced feelings of depression and anxiety.

At T1, physical (*p* < 0.001) and mental (*p* < 0.001) health scores were improved in the VLCKD group. After 12 months, the increases in physical (*p* < 0.001) and mental (*p* < 0.001) health scores were maintained in the VLCKD group (Table 5).

No significant improvement of quality of life was reported in LCD, both at T1 and T2 (Table 6).

Adherence to the nutritional regimen was higher in the VLCKD group than in the LCD group (T1 *p*-value = 0.025; T2 *p*-value = 0.004). In fact, patients in the VLCKD group showed a higher satisfaction than LCD patients, due to rapid weight-loss, reduced sense of hunger and the general feeling of well-being during the ketosis phase. This may help to explain the scarce effects of the LCD on weight loss at both points of observation. The scores obtained in adherence tests at T1 and T2 in VLCKD and LCD groups are reported in Table 7. No adverse reactions to replacement meals were reported among the patients in the VLCKD group.

## 4. Discussion

In this retrospective study we demonstrated that a VLCKD determines a rapid and significant improvement in metabolic parameters, anthropometric measures and quality of life in patients with obesity and T2DM. These improvements were maintained at 12 months, and were accompanied by a drastic reduction in the requirement for T2DM medications and, in some cases, by T2DM remission. Despite having a similar metabolic profile and lower BMI at the beginning of the study, the subjects in the LCD group did not experience the improvements in metabolic and anthropometric parameters observed in the VLCKD group.

The patients who followed a VLCKD showed a higher adherence to the prescribed nutritional regimen, compared to patients in the LCD group, probably due to the rapid weight loss, feeling of satiety and well-being, and user friendliness of the meal-replacement protocol, experienced by the former group.

The lower adherence of patients to the LCD regimen may explain the scarce effects on weight loss and metabolic parameters observed at both 3 and 12 months, and represents a limitation of the study.

Due to the threatening increase in the prevalence of T2D and obesity [30], effective strategies are necessary for weight loss and maintenance [31,32,33]. Although bariatric surgery is an effective treatment option for patients with T2DM and obesity, its invasiveness, high costs, long waiting lists and potential complications limit its widespread use. Therefore, pharmacological and lifestyle-based management represent a valuable option for the majority of patients with diabesity [34]. However, dietary regimens are often characterized by limited efficacy in weight loss and poor adherence in the majority of patients. Alternative dietetic strategies have been introduced in order to obtain greater weight loss and higher adherence. Several studies have shown that a marked improvement in glycemic control can be achieved through a drastic caloric restriction using a very-low-calorie diet [35,36,37]. In particular, VLCKD is a valid approach in people affected by obesity and T2DM [15,16,17,38], since it promotes satiety, rapid weight loss, and muscle sparing. Importantly, recent studies investigated the role of VLCKD in short-term remission of T2DM [39,40,41]. In a recent meta-analysis, 13 studies were included to investigate the effects of KD on T2DM [42]. They found that after a Ketogenic Diet, the average reduction in fasting glucose was 1.29 mmol/l (95% CI: −1.78 to −0.79), whereas the average reduction of HbA1c was 1.07% (95% CI: −1.37 to −0.78), which is considered a valuable therapeutic goal in the management of diabetes [43]. Unfortunately, the limitation of most of these studies is the absence of long-term data.

In this study we demonstrated the beneficial effects of a VLCKD in the management of diabetes over 12 months, with a decrease or even suspension of antidiabetic treatments observed at the end of the study. In the LCD group, the number of antidiabetic medications was increased in almost 50% of the patients, suggesting a strict relationship between failure to adhere to a nutritional regimen and increased need of pharmacological intervention.

In addition, this study investigated changes in eating behavior and quality of life during nutritional therapy. The TFEQ, a common and well-established self-rating questionnaire, measures three aspects of eating behavior: “cognitive restraint”, “disinhibition” and “hunger”. In the VLCKD group, the uncontrolled eating and emotional eating scores decreased during the ketosis phase, in keeping with the established role of ketone bodies in suppressing hunger. Likewise, the poor adherence to the LCD was probably due to an increased appetite in the LCD group. Importantly, the continuous sense of hunger or lack of a satiety effect of most dietary regimens favors erroneous eating behaviors and weight gain.

Between T1 and T2, VLCKD patients showed a mild increase in uncontrolled eating and emotional eating scores and a reduction in cognitive restraint scores, probably due to the reintroduction of carbohydrates, inducing a lower sense of satiety and increased appetite.

Finally, the mean score of each of the SF36-items was significantly increased in the VLCKD group throughout the study, suggesting a general improvement of the quality of life (reduction in physical pain, improvement of daily activities and better mental health). Conversely, the perception of quality of life remained globally unchanged in the LCD group.

No clinically significant side effects were observed in patients treated with VLCKD, apart from mild constipation and dizziness in the first week of the regimen. Kidney and liver function parameters remained unchanged during the diet protocol, in accordance with the reported safety characteristics of this regimen [12].

Meaningful limitations exist in this study. First of all, the number of patients is small to draw solid conclusions. Importantly, the VLCKD and LCD groups differed in mean baseline BMI and anthropometric measures: such differences may be explained by the fact that diabetic patients affected by more severe degrees of obesity were more likely to accept and to adhere to a VLCKD regimen. Notably, in this study all patients were free to choose to follow a VLCKD or LCD nutritional program. This may represent a bias of the study; indeed, patients who chose the LCD protocol were probably less motivated to adhere to a strict nutritional regimen and actually showed less inclination to change their eating habits. In fact, adherence to the nutritional regimen was significantly lower in the LCD group, which was not unexpected, since in clinical practice diabetic patients often show a poor adherence to nutritional interventions and lifestyle modifications.

## 5. Conclusions

The present work is a retrospective observational study, and should be considered as a real-world evidence study, which evaluated in a hospital clinical practice setting the effectiveness of different diet strategies for the management of diabetic patients. The study confirms that VLCKD represents a safe and effective tool in the management of obesity and T2DM, also in accordance with the American Diabetes Association recommendations [38]. Due to its beneficial metabolic effects, VLCKD should be regarded as a safe and effective strategy of lifestyle intervention and metabolic rehabilitation in properly selected and motivated patients affected by obesity and T2DM [16], which can lead to a decrease, or even suspension, of pharmacological therapy, potentially causing remission of the disease.

## Figures and Tables

**Table 1:** Baseline characteristics (± SD) of very-low-calorie ketogenic diet (VCLKD) and low-calorie diet (LCD) group.

Parameters	VLCKD (*n* = 15)	LCD (*n* = 15)	*p*-Value
Age (years)	60.5 ± 10.2	64.4 ± 8.8	0.271
Sex	Female 7 (47%), Male 8 (53%)	Female 7 (47%), Male 8 (53%)	NA
Weight (kg)	111.6 ± 19.8	91.6 ± 18.7	**0.008**
BMI (kg/m^2^)	39.5 ± 6.0	32.2 ± 4.3	**0.001**
WC (cm)	118.2 ± 9.0	103.1 ± 11.6	**0.000**
HC (cm)	119.4 ± 14.9	105.5 ± 9.3	**0.005**
WHR	1.00 ± 0.11	0.97 ± 0.05	0.452
BPsys (mmHg)	143.2 ± 16.3	139.6 ± 13.1	0.512
BPdias (mmHg)	85.4 ± 6.9	81.2 ± 7.0	0.105
Glycemia (mg/dL)	118.2 ± 18.8	129.3 ± 33.6	0.273
HbA1c (%)	6.6 ± 0.84	6.7 ± 0.69	0.642
Tot Chol (mg/dL)	203.4 ± 35.0	196 ± 26.7	0.518
HDL chol (mg/dL)	42.4 ± 13.6	42.3 ± 8.6	0.975
LDL chol (mg/dL)	126.0 ± 38.3	117.7 ± 29.2	0.509
Trig (mg/dL)	188.2 ± 36.4	179.8 ± 20.3	0.444
Creatinine (mg/dL)	0.89 ± 0.25	0.83 ± 0.20	0.517

BMI: Body Mass Index; WC: Waist circumference; HC: Hip circumference; WHR: Waist–hip ratio; BPsys: systolic blood pressure; BPdiast: diastolic blood pressure; Tot Chol: total cholesterol; HDL chol: high-density lipoprotein cholesterol; LDL chol: low-density lipoprotein cholesterol; LCD, low-calorie diet; Trig: triglycerides; VLCKD, Very-low-calorie ketogenic diet. All values are presented as mean ± standard deviation. Differences were considered statistically significant when *p* was <0.05. NA, not applicable. Significant *p* values are highlighted in bold.

**Table 2:** Characteristics and pharmacological treatment at baseline and after 12 months in VLCKD and LCD groups.

**Characteristics**	**VLCKD**		**LCD**	
	**Baseline (T0)**	**After 12 Months (T2)**	**Baseline (T0)**	**After 12 months (T2)**
Subjects	15		15	
Men	8		8	
Women	7		7	
Diabetes Duration (years)	2.53 ± 1.19		2.47 ± 1.36	
**Pharmacological Treatment**	**VLCKD**		**LCD**	
Diet	1	4	0	0
Metformin + Diet	5	11	15	8
Metformin + Sulphonylurea	2	0	0	0
Metformin + GLP-1 agonists	2	0	0	6
Metformin + SGLT2 inhibitors	3	0	0	1
Metformin + DPP4 inhibitors	2	0	0	0

**Table 3:** Anthropometric and biochemical parameters (±SD) at baseline, T1 and T2 in VLCKD and LCD groups.

Parameters	VLCKD T0	VLCKD T1	*p* T0-T1	VLCKD T2	*p*T0-T2	LCD T0	LCD T1	*p*T0-T1	LCD T2	*p*T0-T2
Weight (kg)	111.6 ± 19.8	102.1 ± 16.1	**0.000**	98.7 ± 13.4	**0.000**	91.6 ± 18.7	91.3 ± 19.6	0.706	90.5 ± 19.5	0.623
BMI (kg/m^2^)	39.5 ± 6.0	35.9 ± 5.3	**0.000**	34.8 ± 4.04	**0.001**	32.2 ± 4.3	29.7 ± 8.6	0.252	32.0 ± 4.5	0.442
WC (cm)	118.2 ± 9.0	113.8 ± 7.5	**0.000**	114.5 ± 6,68	**0.002**	103.1 ± 11.6	102.4 ± 11.5	0.274	102.4 ± 12.0	0.396
HC (cm)	121.6 ± 16.4	118.1 ± 15.2	**0.008**	117.1 ± 13.9	**0.006**	105.2 ± 10.6	104.1 ± 10.5	0.173	104.2 ± 10.2	0.141
WHR	1.00 ± 0.13	0.98 ± 0.13	0.276	0.99 ± 0.13	0.399	0.98 ± 0.04	0.98 ± 0.05	0.493	0.98 ± 0.04	0.169
SysBP (mmHg)	143.2 ± 16.3	134.6 ± 10.7	**0.001**	129.4 ± 10.0	**0.002**	136.4 ± 12.3	133.4 ± 12.9	**0.024**	138.8 ± 7.9	0.486
DiasBP (mmHg)	87.5 ± 5.59	82.7 ± 6.8	0.094	76.5 ± 9.3	**0.007**	80.8 ± 7.78	78.6 ± 7.60	0.471	85.7± 9.0	0.307
Tot Chol (mg/dL)	205.7 ± 35.1	178.5 ± 35.7	**0.001**	160.1 ± 40.1	**0.001**	187.3 ± 21.0	174.9 ± 43.9	0.475	177.5 ± 33.4	**0.072**
HDL Chol (mg/dL)	46.3 ± 14.25	45.8 ± 14.23	0.736	40.0 ± 10.4	0.171	45.2 ± 9.7	40.0 ± 7.2	0.285	48.6 ± 7.4	**0.065**
LDL Chol (mg/dL)	130.8 ± 31.0	105.5 ± 33.3	**0.010**	85 ± 35.5	0.219	103.4 ± 21.3	98.0 ± 38.6	0.758	100.7 ± 30.1	0.247
Glycemia (mg/dL)	118.2 ± 18.8	103.2 ± 20.0	**0.024**	105.2 ± 7.0	**0.009**	132.0 ± 33.1	123.8 ± 36.9	0.198	127.5 ± 33.4	0.071
HbA1c (%)	6.6 ± 0.84	5.9 ± 0.71	**0.002**	6.2 ± 0.66	**0.002**	6.8 ± 1.00	6.4 ± 0.49	0.130	6.5 ± 0.55	0.273
Creatinine (mg/dL)	0.89 ± 0.25	0.85 ± 0.17	0.172	0.87 ± 0.5	0.527	0.83 ± 0.21	0.85 ± 0.18	0.487	0.81 ± 0.17	0.798
AST (U/L)	29.9 ± 8.79	28.9 ± 6.47	0.512	26.7 ± 5.19	0.057	N	N	N	N	N
ALT (U/L)	30.4 ± 16.45	29.1 ± 13.8	0.389	25.6 ± 7.56	0.141	N	N	N	N	N

BMI: Body Mass Index; WC: Waist circumference; HC: Hip circumference; WHR: Waist–hip ratio; SysBP: systolic blood pressure; DiasBP: diastolic blood pressure. Tot Chol: total cholesterol; HDL Chol: high-density lipoprotein cholesterol; LDL Chol: low-density lipoprotein cholesterol; LCD, low-calorie diet; VLCKD, Very-low-calorie ketogenic diet. N, not available. Statistically significant changes in weight and metabolic control at 3 months (T1) and 12 months (T2) versus baseline were assessed with *t*-paired test and Bonferroni post hoc analysis. The level of significant difference was set to *p* < 0.0025, corresponding to a 5% first type error. All values are presented as mean ± SD. Significant *p* values are highlighted in bold.

**Table 4:** Mean differences (±SD) between baseline (T0), 3 months (T1) and 12 months (T2) in VLCKD and LCD groups.

Parameters	∆ T0-T1 (Mean ± SD)		*p*-Value	∆ T0-T2 (Mean ± SD)		*p*-Value
	VLCKD	LCD		VLCKD	LCD	
Weight	9.51 ± 7.13	0.31± 3.08	**0.000**	12.93 ± 8.84	0.58 ± 4.18	**0.000**
BMI	3.57 ± 2.16	0.41 ± 1.38	**0.000**	4.76 ± 2.78	0.19 ± 1.53	**0.000**
WHR	0.01± 0.03	0.00± 0.01	0.609	0.01 ± 0.04	0.00 ± 0.02	0.638
BPsys	8.53 ± 7.62	−2.20 ± 16.81	**0.032**	13.73 ± 14.34	−2.40 ± 10.44	**0.005**
BPdias	9.33 ± 11.54	2.87 ± 9.49	0.104	9.33 ± 11.54	−3.80 ± 14.25	**0.018**
HbA1c (%)	0.69 ± 0.65	0.42 ± 1.01	0.533	0.61± 0.54	0.13 ± 0.76	**0.070**

Significant *p* values are highlighted in bold. HbA1c plasma levels are expressed as a percentage of total hemoglobin.

**Table 5:** Mean score (± SD) value of the 36-Item Short Form Health Survey questionnaire (SF-36) test at baseline, T1 and T2 in VLCKD group.

SF-36	VLCKDT0	VLCKDT1	*p*-Value T0T1	VLCKDT2	*p*-Value T0T2
PF	43.4 ± 14.2	56 ± 12.4	**0.005**	69.6 ± 9.12	**0.000**
RP	41.4 ± 14.8	61.6 ± 11.7	**0.000**	72 ± 5.4	**0.000**
BP	41.7 ± 14.8	59.3 ± 12.7	**0.001**	62.8 ± 12.4	**0.000**
GH	49.9 ± 47.5	66 ± 11.7	**0.000**	73.6 ± 7.4	**0.000**
VT	47.5 ± 14.0	68.3 ± 11.7	**0.001**	68.7 ± 10.6	**0.001**
SF	45.6 ± 10.7	65.7 ± 8.0	**0.000**	68.8 ± 10.1	**0.000**
RE	57.3 ± 9.2	69.8 ± 11.4	**0.005**	72.2 ± 8.5	**0.000**
MH	51.6 ± 12.1	66.4 ± 12.4	**0.000**	70.2 ± 9.7	**0.002**

PF: physical functioning, RP: role—physical, BP: bodily pain, GH: general health, VT: vitality, SF: social functioning, RE: role—emotional, MH: mental health. Significant *p* values are highlighted in bold.

**Table 6:** Mean score (± SD) value of SF-36 test at baseline, T1 and T2 in LCD group.

SF-36	LCDT0	LCDT1	*p*-Value T0T1	LCDT2	*p*-Value T0T2
PF	59.6 ± 13.3	65.4 ± 13.4	0.105	60.4 ± 11.7	0.836
RP	64.4 ± 11.4	65.5 ± 12.6	0.079	66.8 ± 10.3	0.060
BP	76.5 ± 12.3	73.8 ± 12.2	0.575	72.8 ± 10.7	0.400
GH	75 ± 9.8	77.8 ± 10.4	0.390	73.9 ± 9.7	0.663
VT	51.8 ± 15.2	54.6 ± 14.4	0.071	53.2 ± 14.6	0.091
SF	54.1 ± 10.4	62.4 ± 9.2	**0.034**	56.6 ± 7.7	0.488
RE	59.6 ± 12.2	61.4 ± 11.6	0.054	61.2 ± 10.0	0.308
MH	61.8 ± 10.8	62.4 ± 8.3	0.078	63.8 ± 8.6	0.056

PF: physical functioning, RP: role—physical, BP: bodily pain, GH: general health, VT: vitality, SF: social functioning, RE: role—emotional, MH: mental health. Significant *p* values are highlighted in bold.

**Table 7:** Adherence diet score (±SD) at T1 and T2 in VLCKD and LCD group.

	Score T1		*p*-Value	Score T2		*p*-Value
	VLCKD	LCD		VLCKD	LCD	
Mean ± DS	10.3 ± 2.4	8.4 ± 2.1	**0.025**	9.9 ± 1.9	7.8 ± 1.6	**0.004**

Significant *p* values are highlighted in bold.

## Data Availability

This is an observational retrospective study, where all anonymized data were obtained from secured electronic medical records. Data sharing is not applicable to this article.
